# Effect of heart rate and blood pressure variability on mortality in Japanese critically ILL patients

**DOI:** 10.1186/2197-425X-3-S1-A535

**Published:** 2015-10-01

**Authors:** A Nishigaki, T Yatabe, Y Takahashi, K Yamashita, M Yokoyama

**Affiliations:** Department of Anesthesiology, Kochi Medical School, Nankoku, Japan

## Introduction

Recently, a number of studies reported that variability in blood glucose levels might influence mortality in critically ill patients [1]. Moreover, heart rate and blood pressure variability have been the recent focus of research in the field of internal medicine [2].

## Objectives

We hypothesized that heart rate and blood pressure variability might affect mortality in patients in an intensive care unit (ICU) and investigated it retrospectively.

## Methods

Our study was approved by the ethics committee of our hospital. We included consecutive patients who were admitted to the ICU between January 2012 and December 2014 in this study. Of these patients, those aged < 20 years and those for whom data on heart rate, blood pressure, APACHE II (Acute Physiology and Chronic Health Evaluation II), and SOFA (Sequential Organ Failure Assessment) scores were unavailable were excluded. Electrocardiograms and non-invasive blood pressure monitoring using a bedside monitor (BSM-9100, Nihon Koden; Tokyo, Japan) were conducted. We defined heart rate and blood pressure variability as the standard deviation (SD) of the 2 variables measured during the first 24 hours after ICU admission. Patients were divided into 2 groups based on their outcome at hospital discharge: the survivor (S) and non-survivor (NS) groups. Patient outcomes were assessed by non-paired t-tests, Chi-squared tests, and a multivariate logistic regression analysis. P values < 0.05 were considered statistically significant.

## Results

A total of 1,874 patients were admitted to the ICU between January 2012 and December 2014. Of these, 1,309 patients were excluded, resulting in a final sample of 565 patients for analysis (521 patients in the S and 44 in the NS group). The median age of the patients was 71 years, and 37% were women. The total mortality rate was 7.8%, and the median hospital stay was 15 days. The APACHE II and SOFA scores were significantly higher in the NS than in the S group (17 ± 7 *vs.* 32 ± 11, p < 0.0001 and 4 ± 3 *vs.* 8 ± 4, p < 0.0001, respectively). A higher proportion of patients in the NS than the S group used catecholamines (50% *vs.* 68%, respectively; p = 0.02). Although blood pressure variability was not significantly different between the 2 groups (11 ± 5 mmHg *vs.* 13 ± 7 mmHg in the S and NS groups, respectively; p = 0.12), heart rate variability was significantly higher in the NS than in the S group (9 ± 6 *vs.* 18 ± 16, p < 0.0001). Both, heart rate variability and APACHEII scores were associated with mortality (odds ratio = 1.06, 95% confidence interval = 1.01-1.11, p = 0.02).

## Conclusions

Our retrospective study suggests that heart rate variability might be associated with increased in-hospital mortality in critically ill Japanese patients.
